# Changing clinical patterns in rheumatoid arthritis management over two decades: sequential observational studies

**DOI:** 10.1186/s12891-016-0897-y

**Published:** 2016-01-27

**Authors:** Aneela N Mian, Fowzia Ibrahim, Ian C Scott, Sardar Bahadur, Maria Filkova, Louise Pollard, Sophia Steer, Gabrielle H Kingsley, David L Scott, James Galloway

**Affiliations:** Department of Rheumatology, King’s College Hospital, Denmark Hill, London, SE5 9RS UK; Department of Rheumatology, King’s College School of Medicine, King’s College London, Weston Education Centre, 10 Cutcombe Road, Denmark Hill, London, SE5 9RJ UK; Department of Rheumatology, University Hospital Lewisham, London, SE13 6LH UK

**Keywords:** Rheumatoid arthritis, Disease activity, Disability, Epidemiology, Clinical outcomes

## Abstract

**Background:**

Rheumatoid arthritis (RA) treatment paradigms have shifted over the last two decades. There has been increasing emphasis on combination disease modifying anti-rheumatic drug (DMARD) therapy, newer biologic therapies have become available and there is a greater focus on achieving remission. We have evaluated the impact of treatment changes on disease activity scores for 28 joints (DAS28) and disability measured by the health assessment questionnaire scores (HAQ).

**Methods:**

Four cross-sectional surveys between 1996 and 2014 in two adjacent secondary care rheumatology departments in London evaluated changes in drug therapy, DAS28 and its component parts and HAQ scores (in three surveys). Descriptive statistics used means and standard deviations (SD) or medians and interquartile ranges (IQR) to summarise changes. Spearman’s correlations assessed relationships between assessments.

**Results:**

1324 patients were studied. Gender ratios, age and mean disease duration were similar across all cohorts. There were temporal increases in the use of any DMARDs (rising from 61 % to 87 % of patients from 1996-2014), combination DMARDs (1 % to 41 %) and biologic (0 to 32 %). Mean DAS28 fell (5.2 to 3.7), active disease (DAS28 > 5.1) declined (50 % to 18 %) and DAS28 remission (DAS28 < 2.6) increased (8 % to 28 %). In contrast HAQ scores were unchanged (1.30 to 1.32) and correlations between DAS28 and HAQ weakened (Spearman’s rho fell from 0.56 to 0.44).

**Conclusions:**

Treatment intensity has increased over time, disease activity has fallen and there are more remissions. However, these improvements in controlling synovitis have not resulted in comparable reductions in disability measured by HAQ. As a consequence the relationship between DAS28 and HAQ has become weaker over time. Although the reasons for this divergence between disease activity and disability are uncertain, focussing treatment entirely in suppressing synovitis may be insufficient.

## Background

Rheumatoid arthritis (RA) management paradigms have changed over the last two decades. There is now greater emphasis on intensive suppressive treatment with greater use of disease modifying anti-rheumatic drugs (DMARDs), including combinations of DMARDs, and the use of biologic treatments when standard DMARDs are insufficient [1]. The impact of treatment can be assessed using standard methods such as the Disease Activity Score for 28 joints (DAS28) [2] and the Health Assessment Questionnaire Disability Index (HAQ) [3]. In recent years ‘Treat to Target’ has provided a management framework. It emphasises that the treatment goal should be achieving remission [4]. Several groups have shown that the frequency of severe disease is declining and there are growing numbers of patients with moderate or low DAS28 scores [5–8]. Despite these findings, studies from the Netherlands have suggested that although disease activity levels have improved over time, levels of disability may actually be increasing [9]. We consider further information is needed about the changing patterns of DAS28 and HAQ scores in RA patients seen in routine treatment settings over the last 20 years. We have undertaken such an evaluation in two adjacent English rheumatology units over two decades examining the changes in treatment, disease activity and disability levels over time in sequential cross-sectional studies.

## Methods

### Setting

Cross-sectional data were collected at four time points from secondary care rheumatology departments at King’s College Hospital and University Hospital Lewisham; these are two adjacent centres in South London. The data were collected prospectively by different trained clinicians using standardised clinical assessments in: (i) 1996-97; (ii) 2001-3; (iii) 2009-10; (iv) 2013-14. The surveys assessed consecutive consenting outpatients attending specialist rheumatology clinics within routine clinical care settings.

### Patients

The hospitals serve ethnically diverse inner city populations with broad ranges of social and financial status. Patients included had a consultant diagnosis of RA by the 1987 criteria of the American College of Rheumatology. Disease duration was calculated from date of rheumatologist diagnosis.

Data for the first three cohorts were collected as surveys undertaken as part of routine care evaluations; these have been previously published [10–12]. The patients were consecutive outpatient attenders; there were no exclusion criteria. Only patients in whom full data for DAS28 components had been collected were studied; this meant excluding 3 % of the previously reported patients. Data for the fourth cohort were extracted from routinely captured clinic attendance electronic patient records; full data was available during the survey period in 520 RA patients from 1200 attenders with inflammatory arthritis.

### Assessments

The data collected comprised demographic details (age, sex and disease duration), information about disease activity including the disease activity score for 28 joints (DAS28) and its component parts with ESR, and the use of synthetic DMARDs, biologic DMARDs and oral steroids. We also collected information about disability scores using the Health Assessment Questionnaire (HAQ), pain (100 mm visual analogue scores) and fatigue (100 mm visual analogue scores) completed by the patients were collected in the first, second and fourth cohorts (for pain) and second and fourth cohort (for fatigue). HAQ scores, pain VAS and fatigue measures were not collected in the third cohort. Patients were assessed prospectively by medical and nursing staff who had been trained in RA assessments.

### Analysis

Statistical analysis used STATA statistical software (version 13). Age, disease duration, disease activity assessments and other outcomes were described using means and standard deviations (SD) or medians and interquartile ranges (IQR) for non-normal data. DAS28 category proportions were given as raw figures and percentages. DAS28 remission was defined as a score of <2.6, low disease activity 2.6 – 3.2, moderate disease activity 3.2 – 5.1, sever disease >5.1. Spearman’s correlations were used to assess relationship between the DAS28 and HAQ.

### Local approvals

Collecting data for the first cohort received approval from King’s College Hospital Local Research Ethics Committee; patients provided written informed consent. The third and fourth cohorts utilised data collected through departmental audits. Approval for these collections was obtained from the Trust audit division. All data was anonymised before analysis. No additional data were collected from patients for the purpose of the current study. As the analysis was the secondary use of existing anonymised data our local research and development office approved its use in line with current national research governance arrangements.

## Results

The four cohorts of RA patients seen between 1996 and 2014 ranged in size from 189 to 520 patients. Overall 1324 patients were studied. The groups had similar demographic features (Table 1): 76-80 % were female; their mean ages were 58-60 years; and their mean disease durations were 9.1-10 years.

**Table 1:** Characteristics of patient cohorts

Characteristic	1996-97	2001-03	2009-10	2012-14
	*n = 189*	*n = 310*	*n = 304*	n = 520*
Female gender, n (%)	140 (74 %)	237 (76 %)	244 (80 %)	413 (79 %)
Age, mean (SD)	59 (14)	60 (13)	59 (15)	58 (15)
Disease duration in years, median (IQR)	8 (13)	9 (10)	10 (9)	10 (9)
Any DMARD, n (%)	115 (61 %)	199 (64 %)	242 (80 %)	420 (87 %)
Combination DMARDs, n (%)	1 (1 %)	31 (10 %)	65 (21 %)	199 (41 %)
Biologic users, n (%)	0	11 (4 %)	53 (17 %)	155 (32 %)
Oral steroids, n (%)	23 (12 %)	70 (23 %)	30 (10 %)	56 (12 %)
DAS, mean (SD)	5.2 (1.6)	4.7 (1.6)	3.8 (1.5)	3.7 (1.6)
Tender joint count, mean (SD)	10.1 (9.2)	7.5 (7.3)	4.1 (5.6)	4.2 (6.2)
Swollen joint count, mean (SD)	7.1 (5.5)	4.8 (4.1)	2.9 (4.2)	1.5 (2.7)
Patient global, mean (SD)	50 (25)	50 (29)	40 (25)	35 (28)
ESR, mean (SD)	35 (26)	33 (27)	26 (21)	19 (21)
HAQ score, median (IQR)	1.30 (1.52)	1.52 (0.79)	Not available	1.32 (0.84)
Pain, Mean (SD)	50 (24)	48 (28)	Not available	44 (28)
Fatigue, Mean (SD)	Not available	50 (28)	Not available	51 (26)

*Legend: SD* standard deviation, *IQR* interquartile range, *n = 484 for DMARD data

Over time more patients received suppressive therapy: in 1996-97 only 61 % of patients received one or more DMARDs; by 2013-14 87 % of patients were receiving them (Table 1). The intensity of suppressive treatment increased over time, with rising numbers of patients receiving combination DMARDs and biologics. Combination DMARD use was 1 % in 1996-97 and increased to 41 % by 2013-14. No biologics were available in 1996-97; their use increased from 4 % in 2001-03, 17 % in 2009-10 and further still to 32 % in 2013-14. In contrast, systemic steroid use was unchanged.

Disease activity fell over time, with the proportion of patients in remission increasing from 8 % in the earliest cohort to 28 % in the most recent (Table 2). In parallel, the proportion with severe disease fell from 50 % in the earliest cohort to 18 % in the most recent, whilst the proportion with moderate disease remained constant. There are differences in the relative contribution of the individual components of DAS28 across disease activity strata (Table 2). Most noticeable are the reductions in swollen joint counts in all strata compared with the increases in patient global scores in most strata. The mean pain VAS improved with each consecutive cohort from 50 in 1996-97, 48 in 2001-03, and 44 in 2013-2014 but mean fatigue scores did not fall (see table).

**Table 2:** Comparison of DAS28 components and disability status across disease activity strata

DAS28 Group	<2.6	2.6 – 3.2	3.2 – 5.1	>5.1
*1996-97 Cohort*				
Number of patients, (%)	15 (8 %)	8 (4 %)	72 (38 %)	94 (50 %)
Tender joint count, mean (SD)	0.7 (1.4)	0.8 (1.4)	4.9 (4.8)	16.4 (8.5)
Swollen joint count, mean (SD)	0.3 (0.9)	4.0 (3.6)	4.5 (3.3)	10.5 (5.1)
Patient global, mean (SD)	14 (7)	26 (16)	42 (20)	64 (21)
ESR, mean (SD)	12 (7)	15 (8)	30 (27)	44 (25)
HAQ, mean (SD)	0.3 (0.4)	0.8 (0.8)	1.0 (0.8)	1.7 (0.8)
*2001-03 Cohort*				
Number of patients, (%)	29 (9 %)	32 (10 %)	115 (37 %)	135 (43 %)
Tender joint count, mean (SD)	0.4 (0.7)	1.3 (1.6)	4.6 (4.2)	12.9 (7.1)
Swollen joint count, mean (SD)	0.8 (1.4)	2.3 (2.6)	3.5 (2.8)	7.5 (4.0)
Patient global, mean (SD)	15 (19)	28 (22)	42 (22)	70 (22)
ESR, mean (SD)	9 (7)	20 (19)	26 (21)	48 (28)
HAQ, mean (SD)	0.7 (0.7)	1.0 (0.7)	1.4 (0.7)	1.9 (0.6)
*2013-14 Cohort*				
Number of patients, (%)	148 (28 %)	67 (13 %)	212 (41 %)	93 (18 %)
Tender joint count, mean (SD)	0.7 (1.6)	1.6 (2.4)	4.7 (4.7)	15.2 (7.9)
Swollen joint count, mean (SD)	0.2 (0.7)	1.0 (1.6)	1.9 (2.3)	5.3 (3.6)
Patient global, mean (SD)	24 (17)	24 (17)	50 (21)	73 (16)
ESR, mean (SD)	9 (7)	22 (17)	26 (18)	34 (23)
HAQ, mean (SD)	0.9 (0.8)	1.2 (0.8)	1.4 (0.8)	1.9 (0.6)

*Legend: SD* standard deviation

In contrast to measures of disease activity, there was no evidence of a fall in disability levels over time. Mean HAQ scores were 1.30 in 1996-97 and 1.32 in 2013-14, with a slightly higher mean HAQ of 1.52 in 2001-03. Comparing disability change over time across the disease activity groups, mean HAQ scores in patients in remission and low disease activity states were higher in 2013-14 compared to 1996-97 (Table 2). Associated with this finding the overall correlation between disease activity and disability became weaker over time; in 1996-97 Spearman’s rho was 0.56, in 2001-03 it was 0.55 and in 2013-14 it was 0.44.

## Discussion

Our consecutive cross-sectional surveys across two collaborating adjacent specialist units in London between 1996 and 2014 showed the changing pattern of RA treatment and disease severity. The key changes were: increases in the use of DMARDs, combination DMARDs and biologics; falls in mean DAS28; and increases in DAS28 remissions. However, HAQ scores were unchanged and correlations between DAS28 and HAQ weakened. The age, gender and disease duration of the four cohorts were similar and consequently changes in DAS28 could not be explained by differences in demographic factors.

There are several possible explanations for the decline in disease severity: RA may be becoming a milder disease; milder cases of RA may be attending outpatient clinics; or RA treatments are becoming more effective. We noted a minor non-significant difference in disease durations in the last two cohorts compared with earlier cohorts (increasing from 8 years to 10 years). We cannot be certain that this did not have some impact on disability levels but it is unlikely to have been substantial.

Our findings suggest a trend towards milder RA. This has been highlighted in studies of inception cohorts of RA patients [13–16]. Similar reduction in disease activity levels have also been described in established RA, and these reductions preceded the introduction of biologic agents [6]. There are several possible explanations for these observed declines in disease severity. Firstly RA may be becoming a milder disease. Secondly, milder cases of RA may be attending outpatient clinics. Finally RA treatments may be becoming more effective. A North American analysis of patients seen over three consecutive decades from 1970 suggested the reduction in disease severity could be explained entirely by adjusting for DMARD exposure, indicating increased treatment accounts for the improvement [8]. Irrespective of why fewer patients have highly active RA, its impact is reflected by declining rates of hand and foot surgery for RA [17].

Studying the DAS28 strata in the different cohorts showed differences over time between subjective and objective components of DAS28. Swollen joint counts fell but patient global responses did not. Composite measures like DAS28 are influenced by many factors in addition to the inflammatory burden of RA. These other factors include comorbidity, especially psychological problems such as depression, and other features of the disease such as pain [18]. The fact that swollen joint counts have fallen so substantially across all disease activity strata implies that the control of synovitis by modern treatment is more effective than would appear to be the case from DAS28 scores alone. The most prevalent RA state under follow up in contemporary practice is moderately active disease, which accounted for 41 % of the most recent cohort, and an evidence-based management strategy is needed for this group.

Throughout the time of the four cross sectional surveys, there was greater emphasis on the use of DMARDs, DMARD combinations and biologics. There has also been an increase in the involvement of specialist nurses. We consider these developments are the most likely explanation of these changes. The greater emphasis on seeing RA patients early may also have influenced the findings. However, the introduction of formal early arthritis clinics in both centres did not occur until after the last survey was completed.

The relationship between disease activity and disability has been studied in detail in historic cohorts, where it was apparent that disease activity was the key driver of disability in early disease, whilst erosive damage became more important in established disease [19]. However, in an era of diminishing erosive damage, the relationship between disease activity and disability may have changed. A longitudinal analysis of the Nijmegen inception cohort actually revealed increasing levels of disability over time [9]. UK data from the Norfolk Arthritis Register (NOAR) revealed similar findings, with increasing levels of reported disability over time, despite reductions in measured disease activity [13]. Of even more relevance is an analysis combining the NOAR cohort with another UK inception cohort that explored HAQ progression over time [16]. Several distinct trajectories were noted; these were predicted by older age, female sex, longer symptom duration, higher DAS28 and lower social class.

Our findings help contextualise the changes in HAQ over time. Whilst we observed an overall reduction in disability, this was possibly driven by a decline in patients with severe disease. In contrast, HAQ scores increased within the lower DAS28 groups. This corresponds to the rise in the subjective components of DAS28, which predictably correlate better with patient reported disability. One explanation for these findings is that background levels of disability might be rising, perhaps due to external factors such as the climbing levels of obesity in the general population. However, this is at odds with data from non-RA populations that have reported reductions in disability in the general population [20]. An alternative explanation is that way patients complete HAQ scores may be changing: patients might report disability differently using the HAQ due to higher expectations. The HAQ tool is also limited by floor and ceiling effects. Irrespective of the reason for the changing pattern of HAQ score, it remains a core metric by which effectiveness of RA treatments are judged [21].

Our study has several strengths. We have reported real time data with no exclusion criteria which is likely to be highly representative of RA patients seen in outpatient clinics at the time of each survey. It was also sufficiently large to provide robust assessments of treatment and disease activity. Our study does however have some limitations. The data were collected at consecutive cohorts was cross sectional and did not capture the same patients. As the data collected were anonymized we were uncertain if some patients contributed to more than one cohort. The assessments were carried out by a number of different physicians over the four time periods and there might have been variations in the way individual clinicians assess joint counts. It is also possible that the pattern of follow up in clinics has changed with RA patients who have milder disease being more frequently. Another possibility is that there may have been changes in the level of background disease activity in patients seen in specialist clinics. These are inherent problems in all studies that evaluate temporal changes in disease activity.

## Conclusions

Over the last two decades the management of rheumatoid arthritis has changed. Patients are treated more intensively with both conventional DMARDs and biologics. Disease activity levels have fallen and more patients achieve remission; however, as only a minority of patients are in remission there is still substantial room for improvement. The failure to substantially reduce disability levels is of some concern. It is possible that focusing on reducing disease activity has less impact on disability than was previously considered. It may be particularly important to focus on reducing tender joint counts and patient global assessments, which may be related to non-inflammatory aspects of RA. Finally, further work is needed to understand how to best manage moderate RA, the phenotype that now makes up the largest burden of clinic attenders.

## Abbreviations

DAS28: Disease activity score for 28 joints
DMARD: Disease modifying anti-rheumatic drug
HAQ: Health assessment questionnaire
IQR: Interquartile range
NOAR: Norfolk arthritis register
RA: Rheumatoid arthritis
SD: Standard deviation
